# Platinum(IV) anticancer therapies and cathepsin B: innovative strategies for overcoming resistance in glioblastoma cells

**DOI:** 10.3389/fcell.2025.1506206

**Published:** 2025-06-04

**Authors:** Claudio Casali, Ludovica Gaiaschi, Enrico Pelloni, Federica Gola, Margherita Cavallo, Gloria Milanesi, Mauro Ravera, Marco Biggiogera, Fabrizio De Luca, Maria Grazia Bottone

**Affiliations:** ^1^ Laboratory of Cell Biology and Neurobiology, Department of Biology and Biotechnology “L. Spallanzani”, University of Pavia, Pavia, Italy; ^2^ Department of Sciences and Technological Innovation (DiSIT), University of Piemonte Orientale “A. Avogadro”, Alessandria, Italy

**Keywords:** glioblastoma, cathepsin B, drug resistance, platinum(IV), apoptosis, mitophagy

## Abstract

Glioblastoma (GBM) is the most frequent and aggressive brain tumor in adults. Due to its heterogeneity, the abundance of altered signaling pathways within the same tumoral mass, its low immunogenicity, and the presence of the blood–brain barrier, standard therapies based on surgical resection, radiotherapy, and chemotherapy result in ineffective tumor removal. For these reasons, the development of new drugs is mandatory to ameliorate patients’ life expectancy and quality of life. Cathepsins are lysosomal proteases involved in several physiological and pathological processes, and they play key roles in modulating cell death and pharmacological resistance. In particular, cathepsin B is a crucial regulatory protein in different types of cell death, and its overexpression contributes to GBM angiogenesis and tumor progression. Octahedral platinum(IV) (Pt(IV))-based prodrugs have already demonstrated improved anticancer efficacy compared to routinely used cisplatin. This work aims to investigate the effects of two such prodrugs—Pt(IV)Ac-POA ((*OC*-6-44)-acetatodiamminedichlorido(2-(2-propynyl)octanoato)platinum(IV)) and DB178 ((*OC*-6-44)-acetatodiamminedichlorido(4,5-dihydroxy-9,10-dioxo-9,10-dihydroanthracene-2-carboxylato)platinum(IV))—on two different glioblastoma cell lines, U251 and T98G, with particular attention to their effects on cathepsin B. The immunocytochemical and biochemical results obtained on the two cell lines highlight the maintenance of basal levels of cathepsin B while efficiently activating programmed cell death mechanisms, as investigated by optical and electronic microscopy. These findings may serve as a valid starting point for further approaches that incorporate cathepsins’ inhibitors to improve therapeutic efficacy and possibly reveal novel pharmacological targets.

## Introduction

Gliomas consist of a heterogeneous group of tumors graded by the World Health Organization based on their microscopic and molecular profiles. Glioblastoma (GBM) is the most aggressive form of high-grade glioma, characterized by a poor prognosis and high mortality rates, with a median patient survival time of less than 15 months. Despite extensive efforts to combat this tumor, GBM remains one of the most lethal neurological malignancies. Its invasive and infiltrative features hinder complete surgical tumor resection, leading to a lack of complete remission (Li et al., 2024; Pichol-Thievend et al., 2024). Furthermore, the tumor microenvironment and the blood–brain barrier, coupled with cellular heterogeneity, the presence of tumor stem cells, and alterations in signaling pathways, significantly contribute to the resistance of GBM to radiotherapy and chemotherapy. The current standard approach involves tumor resection, followed by radiotherapy and adjuvant chemotherapy. However, the inherent features often result in inadequate recovery and disease recurrences (Ferguson et al., 2022; Giordano et al., 2024).

Cisplatin ((SP-4-2)-diamminedichloridoplatinum (II), CDDP) has demonstrated significant clinical efficacy. Once inside the cells and after aquation, it leads to the formation of CDDP–DNA adducts that interfere with DNA replication and transcription. Nonetheless, similar to many other anticancer drugs, CDDP is associated with side effects and drug resistance (Wen et al., 2023), impacting its overall efficacy (Jeon et al., 2021). Consequently, several studies are currently exploring the therapeutic opportunities with platinum-based drugs to leverage their efficacy and overcome the associated limitations. In this field, the analysis of combination therapy using platinum(IV) complexes has shown promising results (Ponte et al., 2023).

**FIGURE 1:**
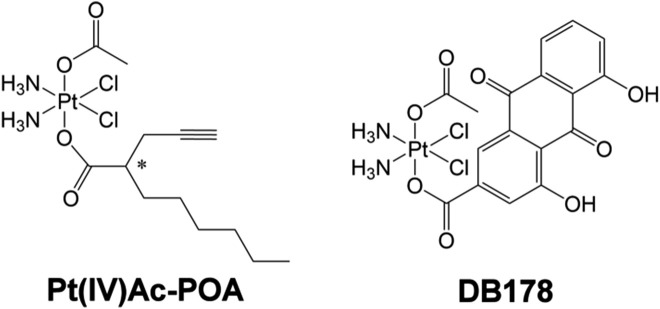
Pt(IV)Ac-POA and DB178. Structural formulas of the octahedral Pt(IV) complexes (*OC*-6-44)-acetatodiamminedichlorido(2-(2-propynyl)octanoato)platinum(IV) [Pt(IV)Ac-POA] and (*OC*-6-44)-acetatodiamminedichlorido(4,5-dihydroxy-9,10-dioxo-9,10-dihydroanthracene-2-carboxylato)platinum(IV) [DB178].

Combination therapy exploits the synergistic effects of two or more compounds to surpass the potential of single-drug treatments. It has been demonstrated that by targeting multiple key pathways, drug combinations may achieve superior efficacy, reduced toxicity, or dosage reduction compared to single drugs (Duarte and Vale, 2022). Octahedral Pt(IV) complexes offer an efficient way to combine a “traditional” Pt(II) moiety (e.g., CDDP) with a second drug in the axial position, having a synergistic or at least additive effect with CDDP. Octahedral Pt(IV) complexes designed in such a way are specifically reduced in hypoxic tumor tissues, resulting in the release of the cytotoxic Pt(II) metabolite alongside the concomitant loss of the second drug in the axial position, a mechanism known as “activation by reduction” (Ravera et al., 2022). The final result is the combination of the selective and stimuli-responsive targeting of cancer cells with the synergistic effect of the platinum moiety and the adjuvant drug (Gabano et al., 2022). Among the octahedral Pt(IV) complexes, the prodrug (*OC*-6-44)-acetatodiamminedichlorido(2-(2-propynyl)octanoato)platinum(IV), also named Pt(IV)Ac-POA (Figure 1), has been reported to exhibit efficient antitumor activity both *in vitro* and *in vivo* (Gabano et al., 2017). Pt(IV)Ac-POA includes, as an axial ligand, the free acid POA—a medium-chain fatty acid and histone deacetylase inhibitor—along with an inert acetate (Ac) group. POA, as a medium-chain fatty acid, enhances the lipophilicity of the drug compared to cisplatin alone, thereby increasing cellular drug uptake (“synergistic cellular accumulation”) (Raveendran et al., 2016; Novohradsky et al., 2015; Ravera et al., 2019). Moreover, the histone deacetylase inhibitor appears to facilitate the formation of Pt(II)–DNA adducts, possibly by reducing the chromatin compaction levels, thus increasing DNA accessibility (Ferrari et al., 2020).

In a similar approach, the platinum moiety has been combined with the naturally occurring rhein (4,5-dihydroxy-9,10-dioxo-9,10-dihydroanthracene-2-carboxylic acid or cassic acid), a molecule known for its anti-inflammatory, anti-oxidant (Yin et al., 2021), and anticancer effects by regulating apoptosis, cell proliferation, migration, and invasion (Henamayee et al., 2020; Zhang H. et al., 2023). This combination constitutes the prodrug (*OC*-6-44)-acetatodiamminedichlorido(4,5-dihydroxy-9,10-dioxo-9,10-dihydroanthracene-2-carboxylato)platinum(IV), also referred to as DB178 (Figure 1). The complexes demonstrated anti-proliferative activity not only higher than that of the parent compounds CDDP and rhein but also higher than the reference drug temozolomide in the same and other human glioblastoma cell lines (Poon et al., 2021; Gabano et al., 2022). Moreover DB178 retained its activity under hypoxia and caused a significant reduction in the motility of both cell lines, which can be related to its ability to inhibit MMP2 and MMP9 matrix metalloproteinases (Gabano et al., 2022).

Cathepsins constitute a class of lysosomal proteases that play a central role in protein degradation; numerous studies have investigated their role in the progression of various tumors; hence, they are being evaluated as potential target candidates for both diagnostic and treatment purposes. In detail, a specific member of the cathepsin family, cathepsin B (CatB), is involved in several physiological and pathological processes, particularly in gliomas, where it is commonly overexpressed, promoting angiogenesis, regulating cell death, and influencing the onset of pharmacological resistance (Ma et al., 2022; Huang et al., 2024). However, the role of CatB in cell survival and its basis of execution exhibit deep variance (Mijanović et al., 2019; Chevriaux et al., 2020); thus, a comprehensive analysis of CatB’s role in GBM is still unavailable, and in particular, no data are available regarding therapies with Pt(IV)-based drugs. Importantly, Pt(IV)-based prodrugs are activated via reduction under hypoxic conditions typical of tumor microenvironments and do not rely on CatB for their activation. Nonetheless, due to its ability to modulate the apoptotic and autophagic pathways, CatB represents a biologically relevant marker when evaluating programmed cell death (PCD) mechanisms triggered by Pt(IV) complexes. This is supported by a substantial body of literature focusing on CatB targeting in GBM. Rempel et al. (1994) first demonstrated increased CatB mRNA expression in glioblastoma cells, correlating it with malignancy and paving the way for emphasizing the contribution of CatB to multiple aspects of GBM progression and patient survival (Norton et al., 2024). Several studies have since incorporated CatB in the characterization of novel therapies, either by developing CatB-responsive programmed brain-targeted drug delivery systems (Jiang et al., 2024), assessing CatB levels following therapeutic interventions, or modulating its expression to observe functional outcomes. For instance, Zhang et al. (2018) reported that irradiation significantly increases CatB expression, and that silencing CatB enhances radiosensitivity in GBM cells by promoting apoptosis through the downregulation of RAD51 and homologous recombination efficiency, hence revealing a possible role for CatB in radioresistance. Despite these findings, data regarding the involvement of CatB in Pt(IV)-based treatments are still lacking, prompting us to include CatB analysis in this study.

For these reasons, this study aims to investigate the effects of the two prodrugs Pt(IV)Ac-POA and DB178 by comparing them with CDDP to evaluate their impact on different molecular pathways. Particular attention will be paid to CatB, considering its central role in tumor progression and its association with other markers indicative of cell proliferation, therapeutic resistance, cellular damage, and cell death. The study will deepen the existing research conducted on the glioblastoma cell line U251 and, concurrently, assess the effects of the drugs on human glioblastoma T98G cells. Particular attention will be paid to the analysis of CatB localization and mitochondria, given the growing number of studies linking these two elements in the context of PCD. Several reports have shown that CatB, especially when released from lysosomes into the cytosol, can trigger a cascade of cell signaling events that ultimately induce the mitochondrial apoptotic pathway through a mechanism involving cytochrome c release and the subsequent promotion of apoptosome assembly (Kavčič et al., 2020; Xie et al., 2023; Zhang Z. et al., 2023).

## Materials and methods

### Cell culture and treatment

The cell lines present in this study were obtained from Sigma-Aldrich, Milano, Italy. Human glioblastoma U251 cells were cultured in 75 cm^2^ flasks or 96-well plates in Eagle’s minimal essential medium supplemented with 10% fetal bovine serum, 2% glutamine, 1% sodium pyruvate, 1% of MEM non-essential amino acid solution, and 100 U/mL penicillin and streptomycin in a controlled 5% CO_2_ humidified atmosphere at 37°C. Human glioblastoma T98G cells were cultured in 75 cm^2^ flasks or 96-well plates in Eagle’s minimal essential medium supplemented with 10% fetal bovine serum, 1% glutamine, 1% sodium pyruvate, 1% of MEM non-essential amino acid solution, and 100 U/mL penicillin and streptomycin in a controlled 5% CO_2_ humidified atmosphere at 37°C. In both cases, cells at a confluence of approximately 80% were treated with CDDP, Pt(IV)Ac-POA, or DB178 for 48 h. For CDDP and Pt(IV)Ac-POA, treatment concentrations were selected according to the literature (Astesana et al., 2021; Gaiaschi et al., 2023a; 2023b; 2024), indicating 40 μM and 10 μM, respectively; for DB178, treatment concentrations were determined based on an MTT (3-(4,5-dimethylthiazol-2-yl)-2,5-diphenyl-2H-tetrazolium bromide) vitality assay test, as subsequently indicated, resulting in 25 μM for U251 and 10 μM for T98G.

### MTT vitality assay

Cells were plated at a density of 5 × 10^3^ cells per well in a 96-well plate with a volume of 100 μL. After 24 h, the culture medium was replaced with a fresh medium containing DB178 treatment. As a control, cells were incubated with fresh culture medium and vehicle (DMSO). Concentrations ranging from 0 μM to 150 μM were tested. After 48 h of exposure, the culture medium was replaced with fresh medium containing a 1:10 dilution of a 5 mg/mL MTT solution in sterile PBS. Following 3 h of incubation at 37°C, tetrazolium salts were dissolved in a volume of 100 μL DMSO per well. Absorbance was measured using an ELx808^TM^ Absorbance Microplate Reader (BioTek Instruments, Inc., Winooski, United States) at 490 nm.

### Immunofluorescence reaction

Immunofluorescence reactions were performed as described in the literature (Gaiaschi et al., 2023b). Cells were plated on coverslips for 48 h to reach approximately 80% confluence and were treated as previously described. Cells were then fixed with 4% formaldehyde for 20 min at room temperature and post-fixed with 70% ethanol at −20°C for 24 h. For the immunofluorescence reactions, samples were rehydrated in 0.2% PBS-Tween 20 , blocked in PBS-Tween 20 (0.2%)–BSA (4%), and then incubated with the primary antibodies (Table 1) diluted in PBS/Tween 20 (0.2%) for 1 h at room temperature. After rinsing with 0.2% PBS-Tween 20, cells were incubated with the corresponding secondary antibodies (Alexa 488- or 594-conjugated anti-mouse, anti-rabbit, or anti-human antibody, Alexa Fluor, Molecular Probes, Invitrogen, Waltham, United States) for 45 min at room temperature. Nuclei were counterstained with 0.1 μg/mL Hoechst 33258 (Sigma-Aldrich, Milano, Italy) for 5 min. Following rinses in PBS, coverslips were mounted with Mowiol and observed using an Olympus BX51 Microscope (Evident Europe GmbH, Hamburg, Germany) equipped with an Olympus MagnaFire camera system and Olympus Cell F software (version 3.1).

**TABLE 1:** List of antibodies used for immunofluorescence immunolabeling. Antigen, antibody name and host, dilution, and reference are reported.

Antigen	Antibody	Dilution	Reference
ACO2	Rabbit polyclonal anti-aconitase 2 (Abcam, Cambridge, United Kingdom)	1:200	ab228923
CatB	Rabbit monoclonal anti-cathepsin B (Cell Signaling Technology, Danvers, United States)	1:200	ab31718
COX4	Mouse monoclonal anti-COX4 (Abcam, Cambridge, United Kingdom)	1:200	ab33985
LC3B	Rabbit polyclonal anti-LC3B (Cell Signaling Technology, Danvers, United States)	1:200	ab2775
Nrf2	Rabbit polyclonal anti-Nrf2 (Abcam, Cambridge, United Kingdom)	1:200	ab31163
Parkin	Rabbit polyclonal anti-parkin (Abcam, Cambridge, United Kingdom)	1:500	ab77924
PINK1	Rabbit polyclonal anti-PINK1 (Abcam, Cambridge, United Kingdom)	1:500	ab23707
Mitochondria	Human autoimmune serum recognizing the 70 kDa E2 subunit of the pyruvate dehydrogenase complex	1:200	Alpini et al. (2012)
Lysosomes	Human autoimmune serum recognizing lysosomal proteinase	1:500	Alpini et al. (2012)

### Transmission electron microscopy sample processing and immunogold labeling

Transmission electron microscopy (TEM) morphological analyses were performed as described in the literature (Casali et al., 2022). After treatments, cells were harvested by mild trypsinization and then centrifuged at 150 *g* for 10 min. For morphology studies, cells were fixed with 2.5% glutaraldehyde in the culture medium for 2 h. Samples were then rinsed with PBS, post-fixed with 1% aqueous OsO_4_ for 2 h at room temperature, pre-embedded in agarose, dehydrated using graded acetone, and finally embedded in epoxy resin (EM-bed812, Electron Microscopy Sciences, Hatfield, United States). Immunogold labeling analyses were performed according to the literature (Casali et al., 2024). Control and treated cells were fixed with 4% paraformaldehyde in the culture medium for 2 h. Following fixation, cells were rinsed with PBS, pre-embedded, incubated in 0.5 M NH_4_Cl for 30 min, dehydrated using graded ethanol, and finally embedded in acrylic resin (LR White, Agar Scientific, Stansted, United Kingdom). In both cases, ultrathin sections (60–80 nm) were cut on a Reichert OM-U3 Ultramicrotome and collected on 300-mesh nickel grids. Grids for immunocytochemical analyses were floated on normal goat serum (NGS) and subsequently incubated overnight at 4°C in a drop of the primary antibody directed against cathepsin B (Table 1). Grids were then rinsed with 0.02% PBS–Tween 20 and PBS, floated on NGS, incubated on a drop of 12-nm colloidal gold particle-conjugated secondary antibody (Jackson ImmunoResearch, West Grove, United States) for 30 min at room temperature, rinsed with PBS, and finally rinsed with dH_2_O. As negative controls, the same experimental procedure was performed using equal volumes without the primary antibody. Grids for both morphological and immunocytochemical analyses were stained with uranyl acetate and lead citrate immediately prior to observation. The specimens were visualized using a JEM 1200 EX II electron microscope (JEOL, Peabody, United States) operating at 100 kV and equipped with a MegaView G2 CCD Camera (Olympus OSIS, Tokyo, Japan). Immunogold labeling was post-processed by false coloring for the sake of readability using Jasc Paint Shop Pro version 7.02.

### Flow cytometry

After treatment, samples were processed for flow cytometry as described by De Luca et al. (2024). In brief, cells were harvested by mild trypsinization with 0.25% trypsin in PBS containing 0.05% ethylene diamine tetraacetic acid (EDTA), rinsed with PBS, and permeabilized in 70% ethanol for 10 min. Following treatment with 100 U/mL RNase A, cells were stained with 50 μg/mL propidium iodide (PI) (Sigma-Aldrich, Milan, Italy). The specimens were analyzed using a BD FACSLyric (Becton Dickinson, Franklin Lakes, United States), and data were analyzed using BD FACSuite Software (v1.3).

### Statistical analysis and data processing

Fluorescence images of the same marker were acquired with a constant exposure time selected based on the control sample, thus ensuring uniform fluorescence intensity for comparison and avoiding bias in the analysis. Optical density was measured using Fiji (Schindelin et al., 2012), according to the established ethical standards for image processing in quantitative analysis. At least 11 different fields were considered for each sample. Statistical analysis was conducted using GraphPad Prism version 5.03 (GraphPad Software, La Jolla, CA, United States). In particular, after evaluating the normality of the parameters, a t-test was selected when comparing two experimental conditions, and one-way ANOVA with Tukey’s *post hoc* test was performed for the comparison of multiple groups. Statistical significance was set at p < 0.05 (*, p < 0.05; **, p < 0.01; ***, p < 0.001). Except where otherwise stated, data were normalized relative to the control, and mean values with standard error of the mean (SEM) are reported in histograms. Tables were assembled using Jasc Paint Shop Pro version 7.02.

## Results

### DB178 treatment concentration evaluation

**FIGURE 2:**
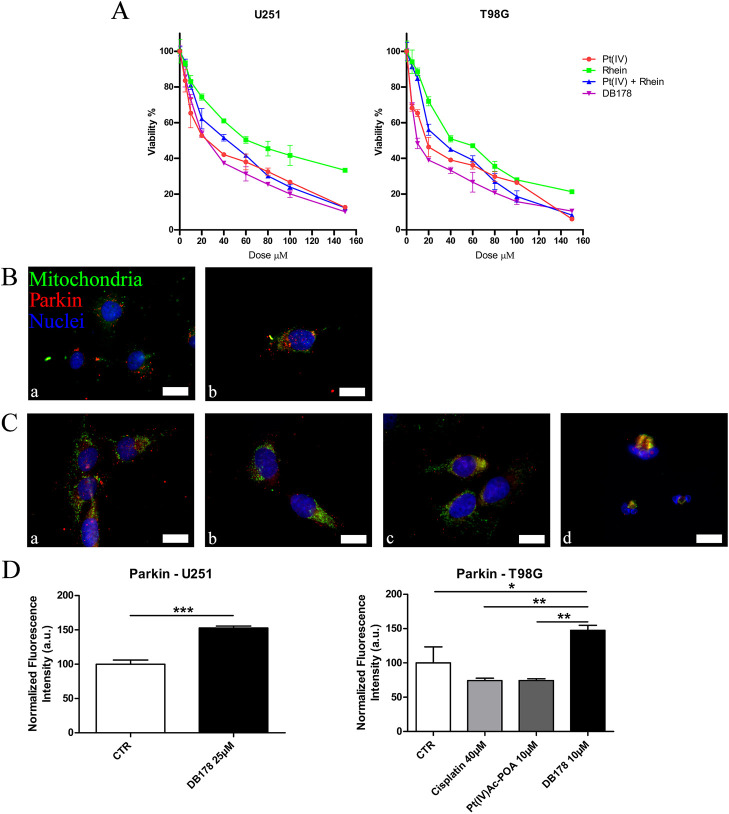
**(A)** MTT assay. Viability curves of U251 and T98G cell lines assessed by the MTT assay after a standard acute exposure of 48 h continuous treatment to increasing concentrations of Pt(IV), rhein, Pt(IV) and rhein, and DB178. Mean values ±SEM are reported; p < 0.0001. (B, C) Immunofluorescent labeling of mitochondria (green) and Parkin (red) in **(B)** U251 (a. CTR and b. DB178 25 µM) and **(C)** T98G cells (a. CTR, b. cisplatin 40 µM, c. Pt(IV)Ac-POA 10 µM, and d. DB178 10 µM). Nuclei were counterstained with Hoechst 33258 (blue). Scale bars, 25 µm. **(D)** Histograms report statistically significant increments in the normalized fluorescence intensity values following DB178 exposure both in U251 and T98G cell lines. *, p < 0.05; **, p < 0.01; ***, p < 0.001.

To determine the optimal concentration of DB178 for further analysis on the cell lines of interest and validate its increased efficacy compared to the separate exposure to the single molecules forming the complex (i.e., Pt(IV) and rhein), a viability MTT test (3-(4,5-dimethylthiazol-2-yl)-2,5-diphenyl-2H-tetrazolium bromide) was performed (Figure 2A). The concentration range of Pt(IV) alone, rhein alone, unconjugated Pt(IV) and rhein, and DB178 tested was from 0 μM (culture medium and vehicle only) to 150 μM. The MTT assay revealed dose-dependent cytotoxicity in both cell lines, with statistically significant increased efficacy of DB178 compared to exposure to separate or contemporaneous unconjugated Pt(IV) and rhein, with a particularly evident effect observed in T98G cells. In detail, the half-maximal inhibitory concentrations of DB178, causing approximately 50% decrease in the number of living proliferating cells, were found to be 25 μM for U251 cells and 10 μM for T98G cells.

As further confirmation of the efficacy of DB178 in reducing the cell proliferation rate, Parkin was investigated. Among the other functions, Parkin is strictly correlated with the regulation of cell cycle progression and is commonly underexpressed in highly proliferating gliomas. Notably, immunofluorescence analysis of Parkin revealed an increment in its optical density following DB178 exposure in both U251 and T98G cell lines, indicating a positive effect in the regulation of the proliferation level (Figures 2B–D).

### The apoptotic pathway is elicited by Pt(IV)Ac-POA: cathepsin B localization and distribution evaluation

**FIGURE 3:**
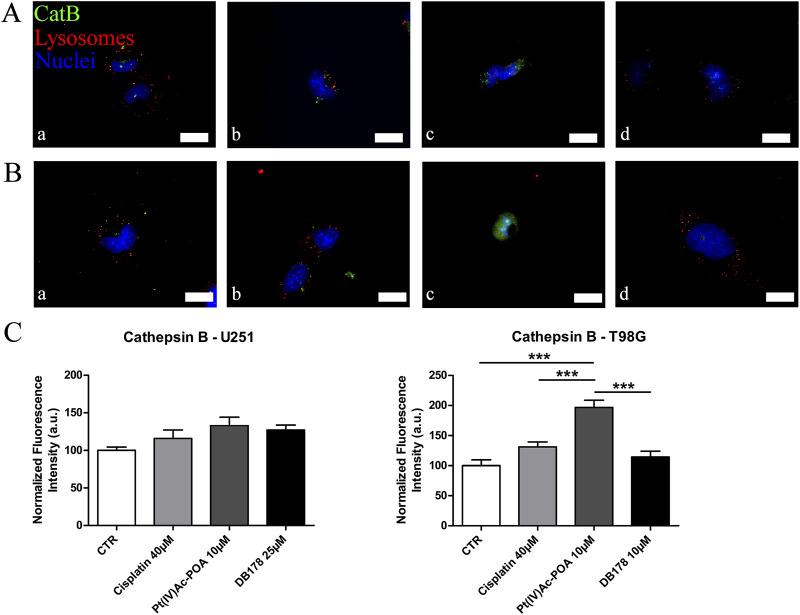
**(A,B)** Immunofluorescent labeling of cathepsin B (green) and lysosomes (red) in **(A)** U251 (a. CTR, b. cisplatin 40 µM, c. Pt(IV)Ac-POA 10 µM, and d. DB178 25 µM) and **(B)** T98G cells (a. CTR, b. cisplatin 40 µM, c. Pt(IV)Ac-POA 10 µM, and d. DB178 10 µM). Nuclei were counterstained with Hoechst 33258 (blue). Scale bars, 25 µm. **(C)** Histograms report maintenance of basal level in U251 cells and a statistically significant increase in the normalized fluorescence intensity values in T98G cells after exposure to Pt(IV)Ac-POA. ***, p < 0.001.

**FIGURE 4:**
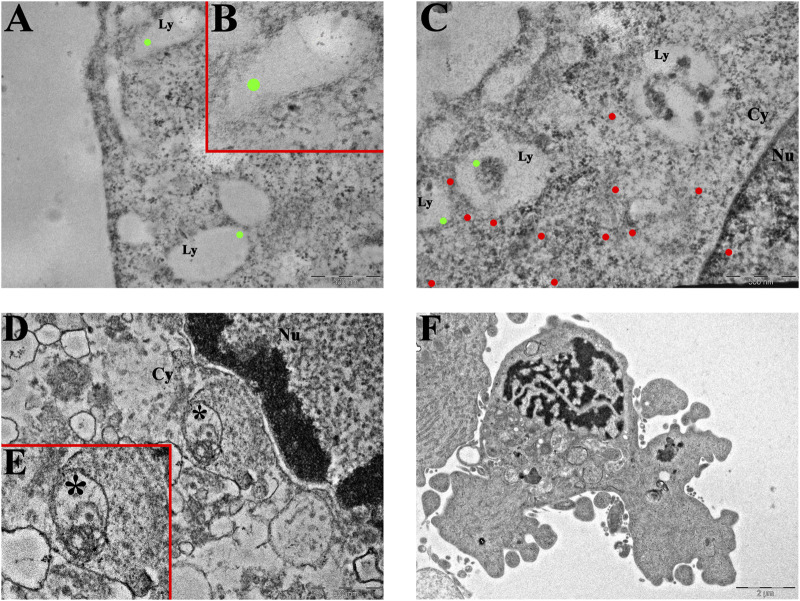
Ultrastructural analysis of T98G cells. **(A–C)** Transmission electron microscopy representative images of CatB immunolabeling in **(A,B)** control and **(C)** Pt(IV)Ac-POA-exposed T98G cells. CatB immunolabeling is confined to lysosomes in control cells (**(B)** inset of **(A)**), while the signal is mainly retrieved in the cytoplasm following treatment. Green dots, lysosomal CatB; red dots, extra-lysosomal CatB. Ly, lysosomes; Cy, cytoplasm; Nu, nucleus. Scale bars, 500 nm. **(D,E)** Representative morphological micrographs of T98G cells representing **(D)** the control cell and **(E)** apoptotic cell, characterized by the presence of a fragmented nucleus and apoptotic bodies, after treatment with Pt(IV)Ac-POA. Scale bars, 2 µm.

Given its multifaceted role, both in contributing to cell death regulation and inducing drug resistance, we evaluated the presence and localization of cathepsin B in U251 and T98G cell lines (Figure 3). CatB immunofluorescent labeling predominantly appeared as spot-like patterns, indicating its confinement within lysosomal vesicles. Upon evaluation of the optical density in both cell lines, our data do not report statistically significant differences in CatB immunolabeling after treatments. Notably, T98G cells treated with Pt(IV)Ac-POA exhibited a significant increase in CatB optical density. Interestingly, the signal appeared to be dispersed in the cytoplasm and nucleus, deviating from the typical confinement to the lysosomal structures. To better characterize this phenomenon, we performed electron microscopy immunogold labeling (Figure 4), which confirmed potential leakages from the vesicles and subsequent cytoplasmic localization of CatB following exposure to Pt(IV)Ac-POA. The ultrastructural analysis allowed the observation of apoptotic cells in this specific condition.

### DB178 treatment is associated with an increment in the mitophagy pathway

**FIGURE 5:**
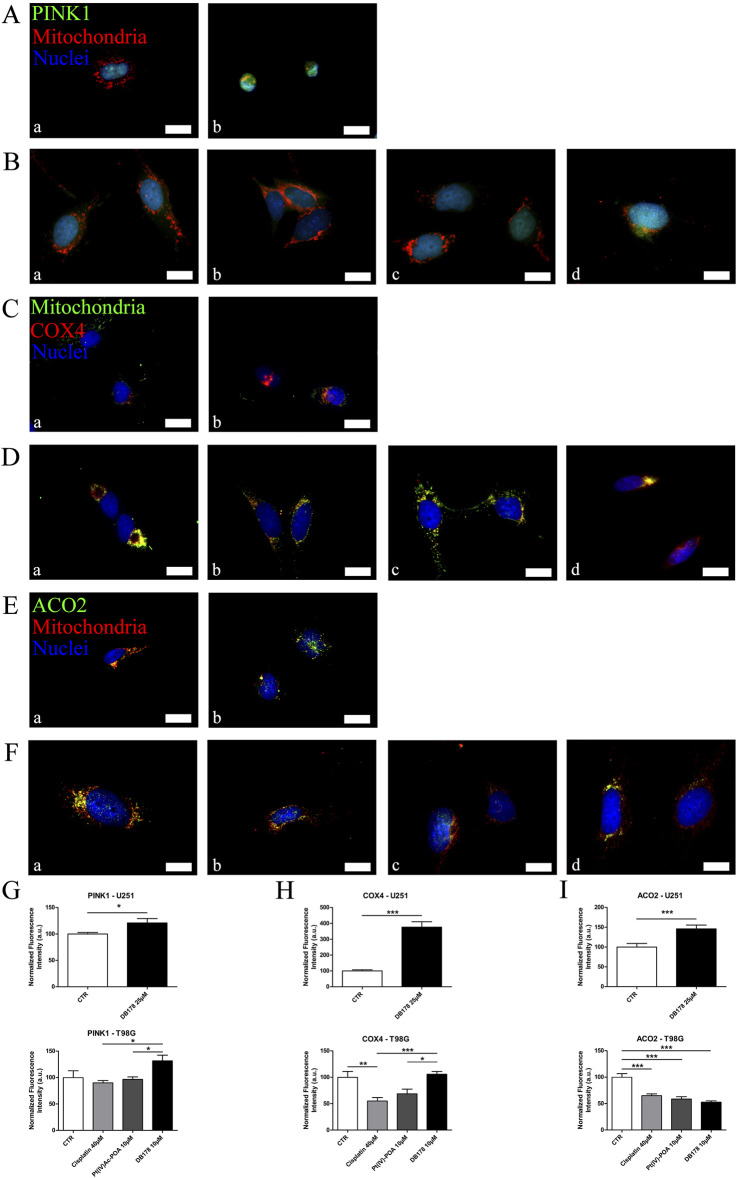
**(A,B)** Immunofluorescent labeling of PINK1 (green) and mitochondria (red) in **(A)** U251 (a. CTR and b. DB178 25 µM) and **(B)** T98G cells (a. CTR, b. cisplatin 40 µM, c. Pt(IV)Ac-POA 10 µM, and d. DB178 10 µM). **(C, D)** Immunofluorescent labeling of mitochondria (green) and COX4 (red) in **(C)** U251 (a. CTR and b. DB178 25 µM) and **(D)** T98G cells (a. CTR, b. cisplatin 40 µM, c. Pt(IV)Ac-POA10 µM, and d. DB178 10 µM). (E, F) Immunofluorescent labeling of ACO2 (green) and mitochondria (red) in **(E)** U251 (a. CTR and b. DB178 25 µM) and **(F)** T98G cells (a. CTR, b. cisplatin 40 µM, c. Pt(IV)Ac-POA 10 µM, and d. DB178 10 µM). Nuclei were counterstained with Hoechst 33258 (blue). Scale bars, 25 µm. **(G)** Histograms report trends of increasing normalized fluorescence intensity values of PINK1 following exposure to DB178 in both U251 and T98G cell lines. **(H)** Histograms report trends of increasing normalized fluorescence intensity values of COX4 following exposure to DB178 in both U251 and T98G cell lines. **(I)** Histograms report statistically significant alterations in normalized fluorescence intensity values of ACO2 in both U251 and T98G cell lines. *, p < 0.05; **, p < 0.01; ***, p < 0.001.

**FIGURE 6:**
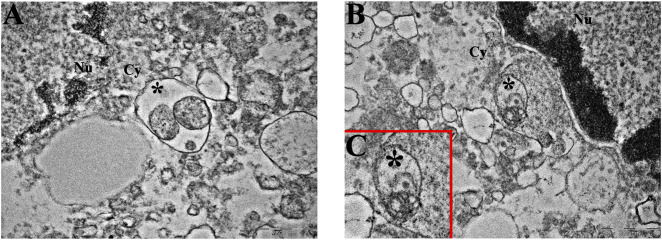
Mitophagy at the ultrastructural level. Representative TEM micrographs showing mitophagic vesicles (asterisk) in **(A)** U251 and **(B, C)** T98G cells following treatment with DB178. (**(C)** inset of **(B)**) Note the remains of the mitochondrial membranous system in the asterisk-labeled vesicle. Cy, cytoplasm; Nu, nucleus. Scale bars, 500 nm.

In addition to its role in regulating the cell cycle, Parkin has been linked to the promotion of mitophagy. In light of the previously described abundance of this protein, especially following DB178 treatment, we deepened the investigation of the potential activation of this pathway by assessing the immunolabeling of PINK1, a mitochondrial enzyme involved in mitophagy events. Our findings highlight a DB178-dependent increase in PINK1 optical density in both cell lines. Furthermore, following DB178 treatment, the PINK1 signal appears more evident in the cytoplasm, particularly at the level of mitochondria. To further characterize this aspect, we assessed the immunolabeling of two other mitochondrial enzymes, COX4 and ACO2, which are involved in regulated cell death and mitophagy. Interestingly, our data highlight that DB178 treatment elicited higher optical density of COX4 in both cell lines, and this was also observed for ACO2 in U251 cells. The results of the double immunolabeling conducted to visualize mitochondria are coherent with these data as the mitochondrial signal after exposure to DB178 is reduced and limited in the cytosolic space compared to the other conditions where the signal appears abundant and more diffuse (Figure 5). As further confirmation, ultrastructural analysis of DB178-treated cells (Figure 6) supports these aspects, revealing various mitophagy events throughout the specimens.

### Pt(IV)Ac-POA and DB178 do not trigger resistance-related markers

**FIGURE 7:**
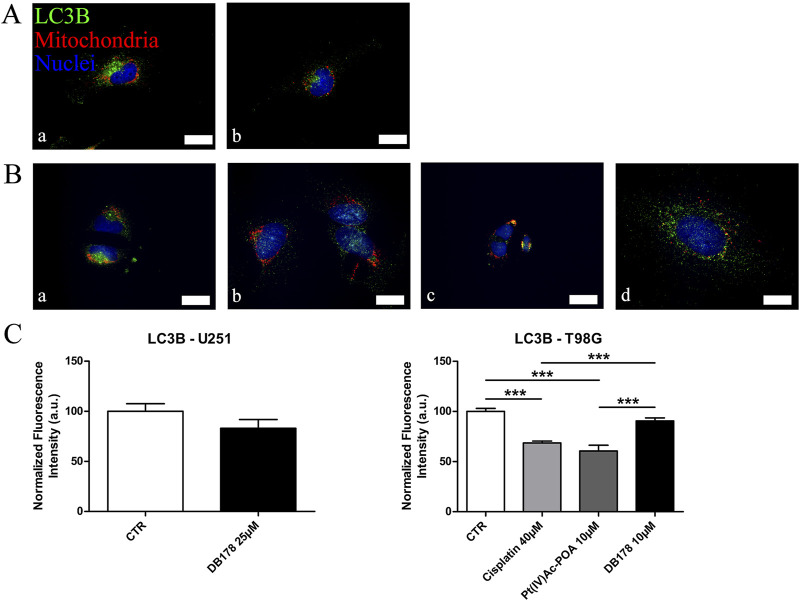
**(A,B)** Immunofluorescent labeling of LC3B (green) and mitochondria (red) in **(A)** U251 (a. CTR and b. DB178 25 µM) and **(B)** T98G cells (a. CTR, b. cisplatin 40 µM, c. Pt(IV)-POA 10 µM, and d. DB178 10 µM). Nuclei were counterstained with Hoechst 33258 (blue). Scale bars, 25 µm. **(C)** Histograms report maintaining low normalized fluorescence intensity values in U251 cells, while statistically significant reductions are evident in T98G cells exposed to Pt(IV)Ac-POA. ***, p < 0.001.

**FIGURE 8:**
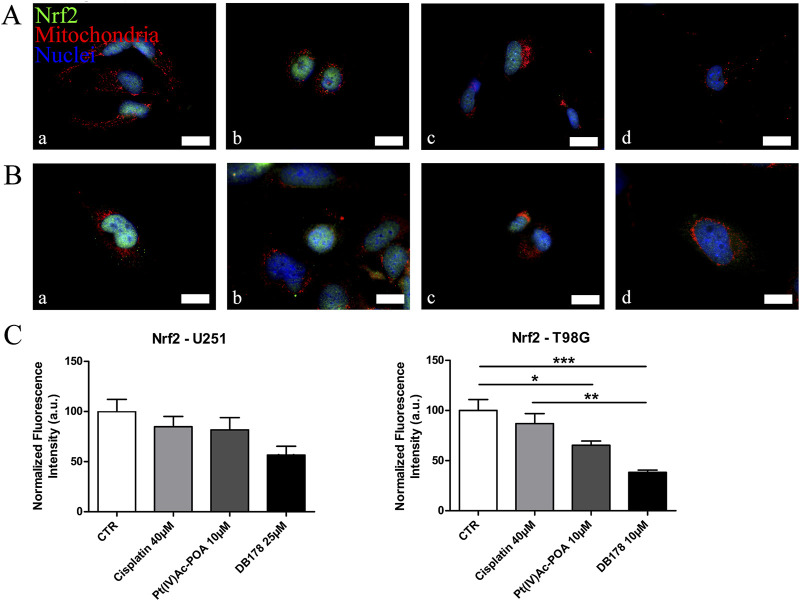
**(A,B)** Immunofluorescent labeling of Nrf2 (green) and mitochondria (red) in **(A)** U251 (a. CTR, b. cisplatin 40 µM, c. Pt(IV)Ac-POA 10 µM, and d. DB178 25 µM) and **(B)** T98G cells (a. CTR, b. cisplatin 40 µM, c. Pt(IV)Ac-POA 10 µM, and d. DB178 10 µM). Nuclei were counterstained with Hoechst 33258 (blue). Scale bars, 25 µm. **(C)** Histograms report trends of reduction in the normalized fluorescence intensity values following Pt(IV) prodrug exposure in both U251 and T98G cells. *: p < 0.05; **: p < 0.01; ***: p < 0.001.

As additional validation of the efficacy of the drugs in avoiding the onset of a prominent pharmacological resistance, we supported the investigation of CatB with an analysis of LC3B, a marker of autophagy and resistance. Immunolabeling of LC3B (Figure 7) revealed that its optical density remained at baseline levels in both U251 and T98G cells following DB178 treatment, with a significant reduction in T98G cells exposed to Pt(IV)Ac-POA. As further confirmation, we evaluated the levels of Nrf2, an enzyme with a protective role that is commonly overexpressed in therapy-resistant glioblastoma cells. Immunolabeling of Nrf2 (Figure 8) delineates the maintenance of baseline levels, with a trend toward its reduction observed in both U251 and T98G cells.

## Discussion

GBM is the most common malignant primary brain tumor, and current therapies only extend overall patient survival and are unsuccessful in providing a definitive cure preventing recurrence phenomena. The development and characterization of novel drugs are essential for the supply of new strategies and therapeutic targets. In this context, octahedral Pt(IV)-based prodrugs are currently being investigated for their potential superior anticancer efficacy. This study aims to characterize the impact of two Pt(IV)-based chemotherapeutic complexes, namely, Pt(IV)Ac-POA and DB178, with specific attention to their effects on markers of proliferation and cell death pathways. These drugs are of particular interest in the context of glioblastoma due to their lipophilic properties, which may facilitate their passage across the blood–brain barrier. Based on previous *in vitro* and *in silico* data, Pt(IV)Ac-POA showed an increased accumulation rate through biological membranes; similarly, promising results were obtained when investigating the lipophilicity of DB178, suggesting that both compounds could the blood–brain barrier more effectively than cisplatin and temozolomide (Gabano et al., 2017; 2022).

PCD is a fundamental physiological mechanism for maintaining cellular homeostasis, and the dysregulation of this process is commonly associated with a variety of human diseases, including cancer. PCD is commonly categorized according to the cellular and molecular findings, and a detailed comprehension of the triggered forms of PCD is vital to characterize the effectiveness of a drug and its putative limitations, particularly regarding the development of resistance (Chen et al., 2024).

In the first part of the work, we focused our attention on DB178. The toxicity profile of this prodrug was assessed through a viability MTT test. This allowed the identification of an IC_50_ value of 25 µM and 10 µM for the U251 and T98G cell lines, respectively. According to data from Cellosaurus^1^ (Bairoch, 2018), the differing sensitivity of T98G and U251 cells to DB178 may be attributed to their distinct TP53 and PTEN mutations, which influence apoptotic regulation and survival pathways. ClinVar^2^ (Landrum et al., 2014) reports that the mutations found in U251 cells are associated with higher oncogenic potential and increased resistance to cell death. Notably, the observed dose-dependent effect was significantly more pronounced when cells were treated with the prodrug, highlighting its superior efficacy compared to the individual components (Pt(IV) or rhein) administered separately. This suggests an adjuvant effect, where the prodrug enhances the activity of its constituents, underscoring the importance of further characterizing the compound, in parallel with the other novel chemotherapeutic prodrug, Pt(IV)Ac-POA, whose synergistic activity has already been proved (Gabano et al., 2017). Unlike what was observed with DB178, Pt(IV)Ac-POA exhibited comparable toxicity in both T98G and U251 cells. Encouragingly, both compounds proved to be more effective at lower doses than CDDP. Although further studies are needed to clarify their specific mechanisms of action and potential mechanisms of resistance, the current findings offer valuable insights into the induction of PCD mechanisms.

Growing evidence supports the idea that cathepsin B is involved in PCD pathways at multiple levels (Liu H. et al., 2023). CatB is the most characterized member of the C1 family of papain-like lysosomal cysteine peptidases. It is synthesized as an inactive pre-proenzyme, glycosylated in the Golgi apparatus, and then transferred to lysosomes (Chou et al., 2023). Although extensive research has been carried out, an unambiguous explanation of the role of CatB under pathological conditions is still not available. For instance, the upregulation of CatB has been associated with increased angiogenic potential, as well as with anti-necroptotic activity and pharmaco- and radioresistance (Mijanović et al., 2019). On the contrary, other studies emphasized the pro-apoptotic activity of CatB due to its role in triggering cytochrome C from mitochondria and subsequent apoptosis (Wang et al., 2023), particularly when released from the lysosomes (Ni et al., 2022). Our data indicate the maintenance of basal CatB levels after treatments in both U251 and T98G cells, suggesting the therapeutic potential by neither stimulating angiogenesis nor promoting resistance onset in surviving cells. The only exception is represented by T98G cells treated with Pt(IV)Ac-POA. To further characterize this aspect, immunogold labeling was employed to localize cathepsin B within specific cellular compartments. This technique has previously been applied to investigate lysosomal proteins with high spatial resolution (Haraguchi et al., 2005; Mahanty et al., 2024). In this condition, CatB immunogold labeling showed elevated labeling of free cytosolic and nuclear CatB compared to the lysosomal counterpart. The release of CatB from lysosomes is known for its important role in determining apoptosis (Yadati et al., 2020; Yoon et al., 2021), which may confirm the positive effect in inducing PCD, as also supported by ultrastructural evidence. The release of CatB into the cytoplasm has been associated with the phenomenon of lysosomal membrane permeabilization, leading to regulated cell death (Reinheckel and Tholen, 2022). Several studies have reported that this release is involved in the activation of intrinsic apoptotic pathways in addition to the extrinsic apoptosis cascade (Pratt et al., 2009). In this context, both genetic manipulation and the use of pharmacological inhibitors have demonstrated that cytosolic CatB contributes to apoptosis through a multilevel mechanism. Notably, CatB can promote the activation of Bid, leading to its translocation to mitochondria and the subsequent release of cytochrome c, which initiates caspase activation and apoptotic cell death. Simultaneously, apoptosis is further favored by the degradation of anti-apoptotic proteins such as Bcl-2, Bcl-xL, Mcl-1, and XIAP (Yadati et al., 2020; Anes et al., 2022). Moreover, CatB has been identified as a key mediator in TNF-α-induced apoptosis (Xie et al., 2023). The presence of these findings in the literature reinforces the relevance of investigating Pt(IV)-based compounds, where the cytosolic relocalization of CatB may indicate effective activation of programmed cell death pathways, alongside the formation of Pt(II)–DNA adducts. Nonetheless, given the dual role of CatB in cancer, further analyses may be necessary to better define the relationship between Pt(IV)-based prodrugs, glioblastoma cell proliferation, and PCD.

For these reasons, we focused our attention on Parkin, a 465 amino acid-long protein encoded by the parkin RBR E3 ubiquitin-protein ligase (PRKN). Parkin, with its E3-ligase activity, has been shown to reduce tumor cell proliferation by blocking access to the G1/S phase of the cell cycle. Parkin expression is reduced in gliomas compared to that in healthy brain tissues, and lower parkin levels are associated with a poor prognosis (Clausen et al., 2024). Pt(IV)Ac-POA has previously been associated with an increase in the Parkin level in U251 cells (Gaiaschi et al., 2023b), while no data were available regarding neither its effect on T98G cells nor DB178 outcome on U251 or T98G cell lines. In this study, we showed that DB178 induced an even greater increase in Parkin levels in U251 cells, demonstrating its potential role in regulating cancer cell progression. Additionally, T98G cells exposed to DB178 exhibit higher Parkin immunolabeling, further supporting the efficacy of this drug compared to standard CDDP treatments.

PINK1, a serine/threonine kinase, acts as a negative regulator of multiple cellular pathways exploited by cancer cells. PINK1 reduces glioblastoma proliferation by controlling aerobic glycolysis, reducing ROS production, and regulating the Warburg effect; furthermore, PINK1 plays a pivotal role in mitophagy (Raimi et al., 2024). Previous studies highlighted an increase in PINK1 levels in U251 cells following Pt(IV)Ac-POA treatment, indicating effective anti-proliferation activity (Gaiaschi et al., 2023b). Our data showed that DB178 treatment also led to a statistically significant increase in PINK1 immunolabeling in U251 cells, supporting the positive effect of this drug. Analogously, we reported a statistically significant increment following DB178 treatment in T98G cells, confirming the drug’s effectiveness on an additional cell line. Moreover, ultrastructural investigations revealed the presence of mitophagy events, consistent with the role of PINK1 in mitochondria quality and abundance control.

To further characterize the effect of drugs on mitochondrial status, we assessed the levels of two different mitochondrial enzymes, COX4 and ACO2. COX4 is the largest regulatory subunit of cytochrome c oxidase, whose overexpression is correlated with the repression of reactive oxygen species (ROS) production, higher respiration rates, mitophagy, and the induction of regulated cell death pathways (Oliva et al., 2022). Pt(IV)Ac-POA has previously been demonstrated not to impact COX4 expression in U251 cells (Gaiaschi et al., 2023b). Interestingly, we found that DB178 correlates with higher levels of COX4. Mitochondrial ACO2 is the Krebs cycle enzyme designated for the conversion of citrate to isocitrate (Kim et al., 2023). Its overexpression has been associated with increased mitochondrial mass and the activation of mitophagy, suggesting an active role in mitochondrial turnover during the quality control process (Jeong et al., 2024). Pt(IV)Ac-POA has already been linked to alterations in ACO2 levels in U251 cells (Gaiaschi et al., 2022). In this study, we demonstrated a similar trend in T98G cells and deregulations of ACO2 following DB178 treatment. The altered levels of ACO2 are associated with cell death induction and mitophagy (Zhu et al., 2023), supporting the activity of Pt(IV) complexes against GBM.

LC3B, an RNA-binding protein and an mRNA decay factor, plays a central role in autophagosome formation during autophagy (Hwang et al., 2022), a process contributing to cell survival and adaptation (Feng et al., 2020; Molina et al., 2022). Recent findings highlighted a worse prognosis in GBM patients with high levels of autophagy-related genes due to increased tumor aggressiveness and therapy resistance (Bashiri and Tabatabaeian, 2023; Danish et al., 2024). Our data indicated that while LC3B levels remained at baseline in U251 cells following treatment with either Pt(IV)Ac-POA (Gaiaschi et al., 2023b) or DB178, a reduction is observed in T98G cells after exposure to Pt(IV)Ac-POA. This decrease may reflect an early block in the autophagic process, which could interfere with the ability of tumor cells to activate survival strategies. Consistently, SQSTM/p62 levels appear globally similar across treatments (Supplementary Material). As a multifunctional adapter protein that links ubiquitinated proteins to the autophagic machinery for degradation—and itself degraded during autophagy (Bjørkøy et al., 2009)—these results support the hypothesis of an incomplete autophagic flux.

Taken together with the other findings, these results suggest that the treatments induce significant mitochondrial damage, triggering mitophagy and the formation of vesicles containing damaged mitochondria, as confirmed by ultrastructural analysis. However, at the time point analyzed, it appears that the autophagic flux was not fully completed (Liu S. et al., 2023). Further investigations, for instance, assessing the colocalization of LC3B with p62 while using reference compounds such as rapamycin and bafilomycin or performing activity and apoptosis assays (e.g. caspase), could represent a valuable approach to clarify this aspect.

Nrf2, a transcription factor with cytoprotective activity, stimulates the expression of genes containing antioxidant response-like sequences in their promoters. Nrf2’s role as a cellular protector applies to both healthy and cancer cells: in normal cells, it protects against oxidative damage and inhibits malignant transformation, whereas in cancer cells, its protective effect may result in resistance to radiotherapy or chemotherapy by preventing apoptosis and cellular death and stimulating drug metabolism and/or drug efflux (Zimta et al., 2019; Ulasov et al., 2022). Cancer cells are known to tolerate moderate levels of oxidative stress, and increased ROS levels contribute to proliferation and evasion from senescence, while excessive exposure to ROS may lead to apoptosis. In this context, the involvement of Nrf2 in NADPH production may prevent excess oxidative damage as a protective mechanism (Pölönen et al., 2019; Cano et al., 2021; Micalizzi et al., 2021). The Nrf2 pathway is overactive in gliomas, contributing to tumorigenesis, cell invasion, and stemness properties (Gumeni et al., 2021; Shahcheraghi et al., 2022; Crisman et al., 2023; Lin et al., 2023). Interestingly, analysis of Nrf2 on T98G cells clearly indicates that Pt(IV) complexes under investigation effectively reduce the levels of this enzymatic cellular protector. This suggests an improved efficiency in limiting resistance acquisition in glioblastoma cells. Although not statistically significant, a similar trend is observed in U251 cells, confirming the potential beneficial effect of these treatments. The combination of these markers supports the hypothesis of reduced development of pharmacological resistance to the assessed Pt(IV) drugs compared to standard cisplatin.

## Conclusion

In conclusion, taken together, these findings underscore the pivotal role of the two investigated octahedral Pt(IV)-based prodrugs in the regulation of GBM proliferation, favoring PCD mechanisms. Pt(IV) complexes are specifically designed to combine a Pt(II) moiety with a second compound in the axial position, allowing for a synergistic or additive effect that is selectively activated in the hypoxic tumor tissue via the established “activation by reduction” mechanism (Ravera et al., 2022). While Pt(IV)Ac-POA incorporates a medium-chain fatty acid to enhance lipophilicity and cellular uptake, along with a histone deacetylase inhibitor to promote the formation of Pt(II)–DNA adduct formation (Ferrari et al., 2020), DB178 is characterized by the presence of rhein, aiming to couple efficient DNA adduct formation with the known anti-proliferative properties of this natural molecule, including its ability to reduce cell motility (Gabano et al., 2022). Although some data on the efficacy of Pt(IV)Ac-POA on U251 glioblastoma cells were already available, this study expanded the current knowledge by confirming its capacity to induce apoptosis in T98G cells. Furthermore, for DB178, no prior data were available on the investigated molecular pathways. Our analysis demonstrated the effectiveness of DB178 against both investigated cell lines. In addition to their efficacy in eliciting PCD mechanisms, both compounds did not significantly induce increases in the detected levels of cathepsin B, further confirming their potential to reduce the possible onset of pharmacological resistance. Furthermore, in the only case in which we found an increase in the cathepsin B signal, immunogold labeling revealed cytosolic relocalization, which aligns with the activation of the apoptotic pathway. Further studies are needed to unravel the complex molecular mechanisms underlying the efficacy of drugs targeting GBM. For instance, the maintenance of basal levels of cathepsin B supports the investigation of Pt(IV)-based prodrugs in combination with cathepsin modulators, which may positively impact the efficacy of the treatments by additional reduction of the proliferative potential of GBM cells. Given the dual role of cathepsin B in either promoting cell proliferation or inducing PCD pathways such as apoptosis, it would be of interest to investigate the effect of the tested prodrugs in combination with cathepsin B inhibitors or, even more intriguingly, functional modulators. This could be pursued in future studies by incorporating commercially available molecules (Siddiqui et al., 2024) and exploring newly synthesized compounds specifically designed for this purpose (Yadav et al., 2024) or through drug repurposing strategies (Alrouji et al., 2024), with the latter two approaches representing rapidly growing areas of research. Such an approach would allow for a deeper understanding of the role of cathepsin B in either sustaining cell proliferation or promoting cell death, depending on its functional modulation. Since tumor recurrence is common with standard therapies, identifying novel and specific molecular targets could support the development of improved drugs that act on multiple signaling pathways, thus improving the efficacy of available treatments and, ideally, taking steps forward in this complex field.

## Data Availability

The original contributions presented in the study are included in the article/Supplementary Material further inquiries can be directed to the corresponding author.

## Footnotes

1
https://www.cellosaurus.org/index.html

2
https://www.ncbi.nlm.nih.gov/clinvar/
