# Supplementary material for: Platinum(IV) anticancer therapies and cathepsin B: innovative strategies for overcoming resistance in glioblastoma cells
Source: Front Cell Dev Biol. 2025 Jun 4;13:1506206. doi: 10.3389/fcell.2025.1506206 (PMC12174597; doi:10.3389/fcell.2025.1506206)
Supplement: Supplementary file 1 [file DataSheet1.pdf]

Platinum(IV) Anticancer Therapies and Cathepsin B: Innovative Strategies for Overcoming Resistance in Glioblastoma Cells

Claudio Casali[1*], Ludovica Gaiaschi[1], Enrico Pelloni[1], Federica Gola[1], Margherita Cavallo[1], Gloria Milanesi[1], Mauro Ravera[2], Marco Biggiogera[1], Fabrizio De Luca[1], and Maria Grazia Bottone[1]

[1]Laboratory of Cell Biology and Neurobiology, Department of Biology and Biotechnology "L. Spallanzani", University of Pavia, 27100 Pavia, Italy.

[2]Department of Sciences and Technological Innovation (DiSIT), University of Piemonte Orientale "A. Avogadro", 15121 Alessandria, Italy.

*Correspondence: claudio.casali@unipv.it ORCID: 0000-0001-8002-8262

Supplementary Materials

This file supplies the original immunogold labeling micrographs, flow cytometry analysis of the U251 and T98G cell lines following the exposure with CDDP, Pt(IV)Ac-POA, and DB178, and the immunofluorescence for SQSTM/p62.

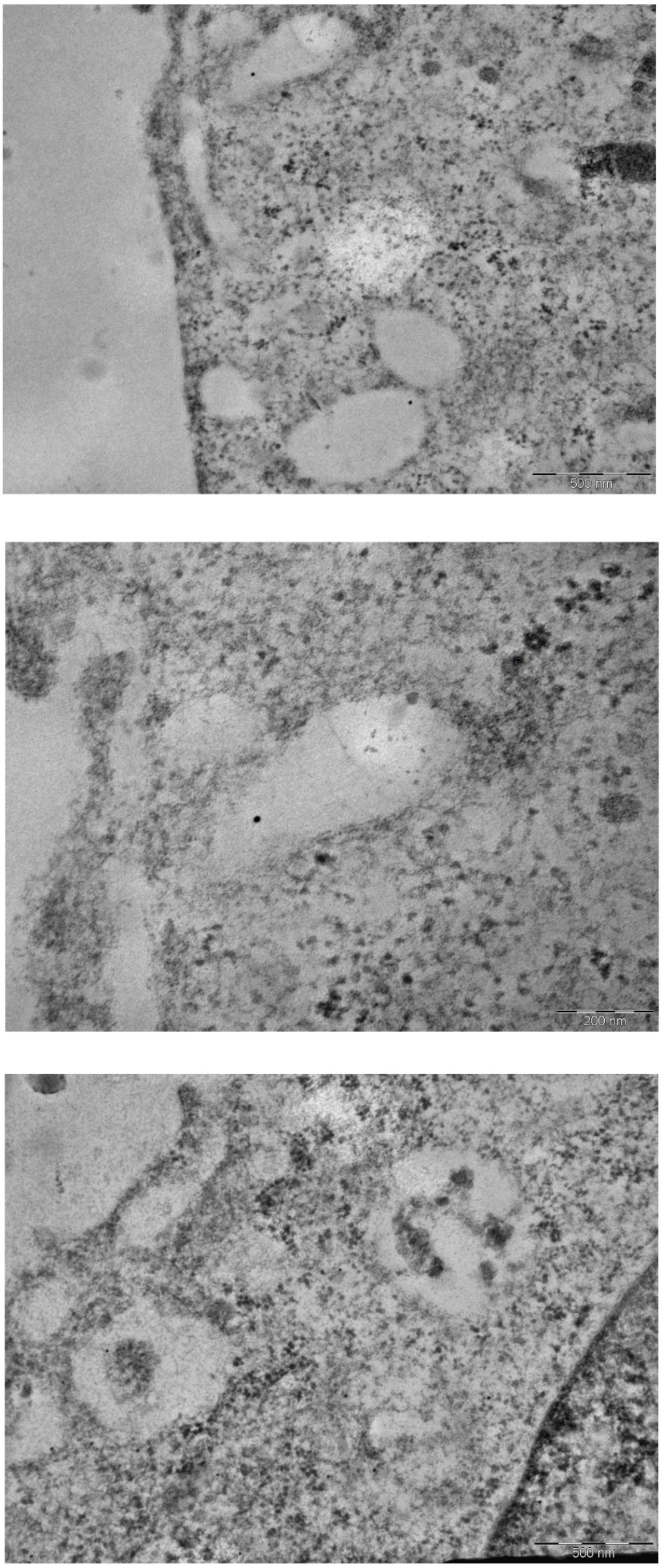

Supplementary Figure 1. TEM immunogold labelling original micrographs, without false-colouring post processing.

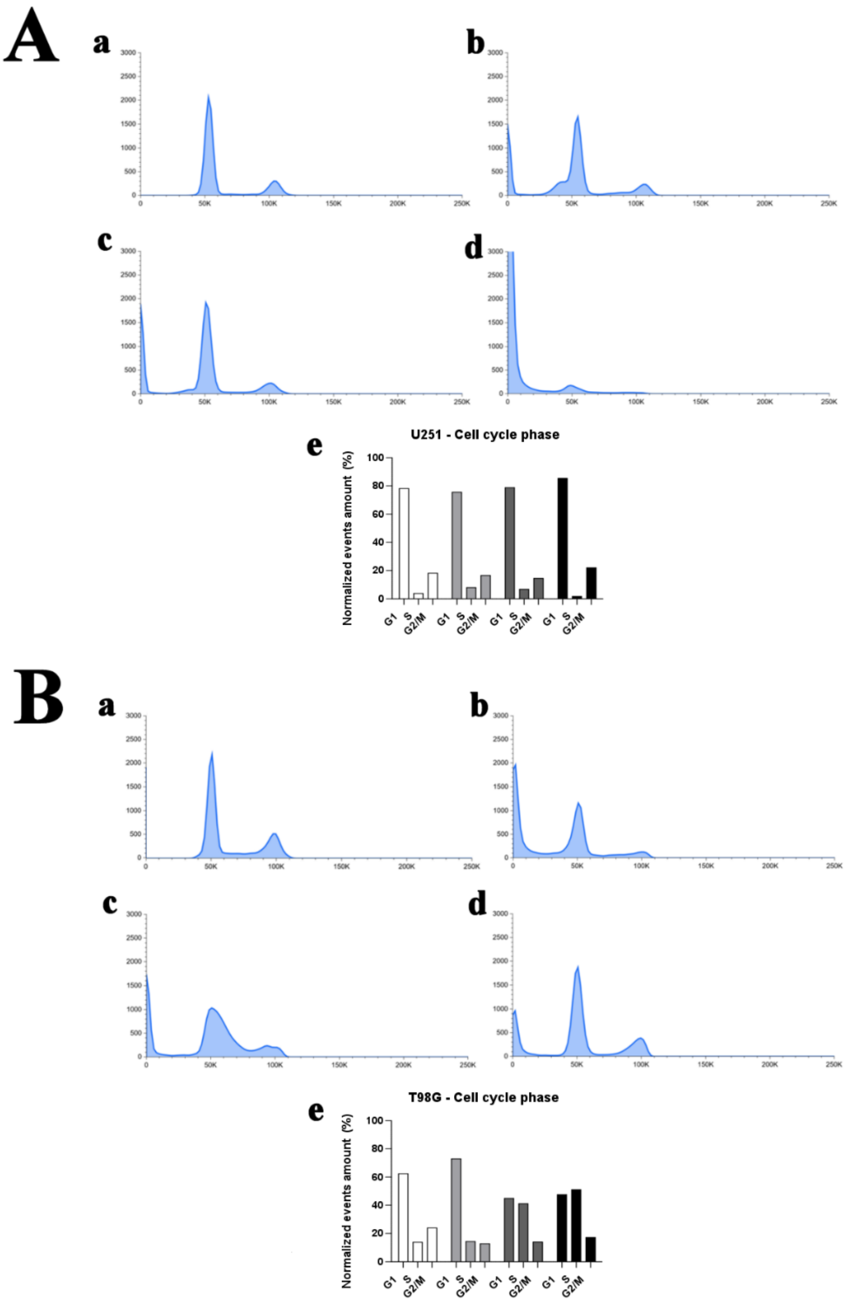

Supplementary Figure 2. Flow cytometry analysis of (A) U251 and (B) T98G cell lines after PI staining, supporting the data concerning cell cycle and the vitality test. (a-d) Cytograms of (a) control, (b) cisplatin 10µM, (c) Pt(IV)Ac-POA 10µM, (d) DB178 25µM (for U251 cells) and 10µM (for T98G cells); (e) representative histograms illustrating the distribution of events among different cell cycle phases (legend of the conditions: left to right control, cisplatin, Pt(IV)Ac-POA and DB178).

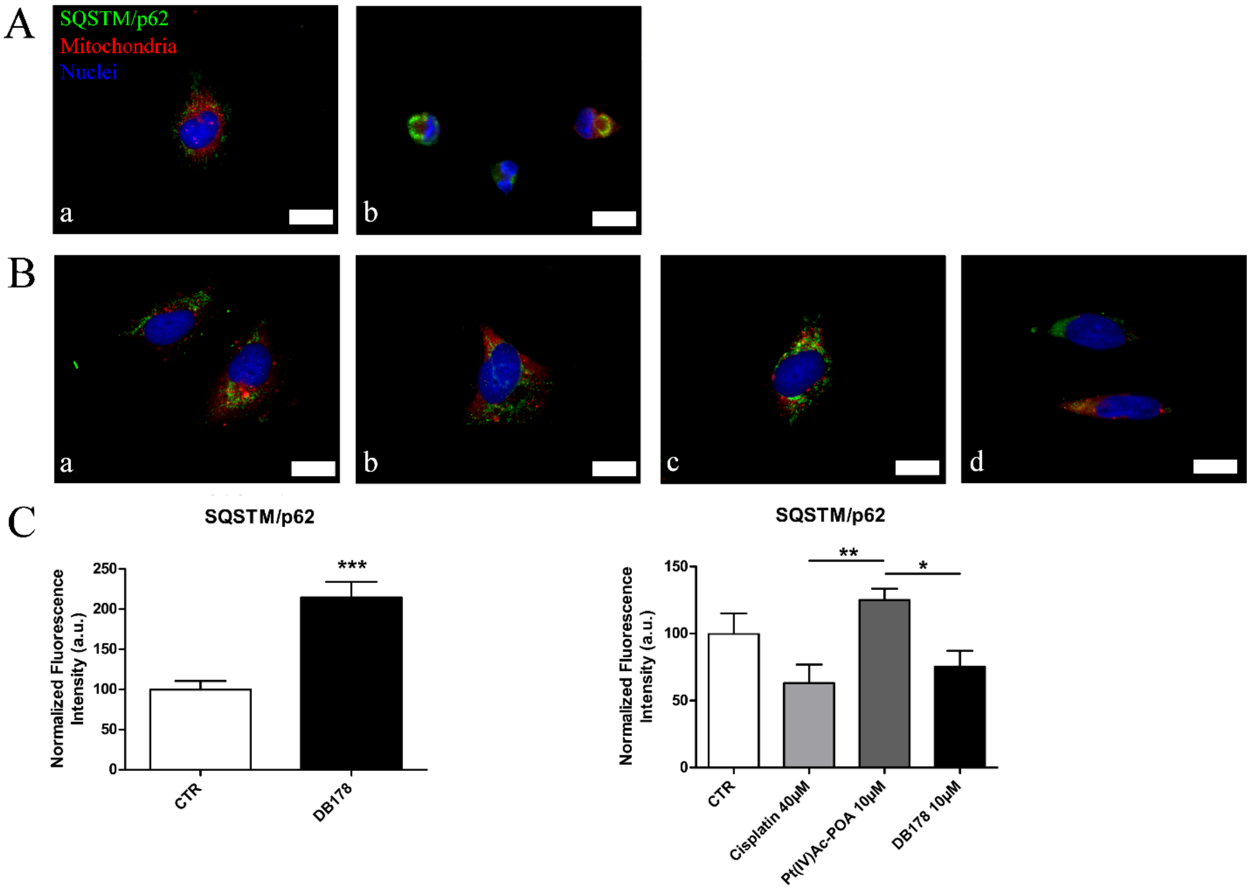

Supplementary Figure 3. A,B. Immunofluorescent labelling of SQSTM/p62 (green) and mitochondria (red) in (A) U251 (a. CTR, b. DB178 25µM) and (B) T98G cells (a. CTR, b. Cisplatin 40µM, c. Pt(IV)-POA 10µM, d. DB178 10µM). Nuclei were counterstained with Hoechst 33258 (blue). Scale bars: 25µm. C. Histograms report no statistically significant reduction in normalized fluorescence intensity values following treatment with Pt(IV)Ac-POA or DB178. DB178 and Pt(IV)Ac-POA induced a statistically significant increase in U251 and T98G cells, respectively. *: $p<0.05$; **: $p<0.01$; ***: $p<0.001$.
